# Treatment outcomes of adjuvant resectional surgery for nontuberculous mycobacterial lung disease

**DOI:** 10.1186/s12879-015-0823-1

**Published:** 2015-02-19

**Authors:** Hyung Koo Kang, Hye Yun Park, Dohun Kim, Byeong-Ho Jeong, Kyeongman Jeon, Jong Ho Cho, Hong Kwan Kim, Yong Soo Choi, Jhingook Kim, Won-Jung Koh

**Affiliations:** Departments of Medicine, Division of Pulmonary and Critical Care Medicine, Sungkyunkwan University School of Medicine, Seoul, South Korea; Department of Thoracic Surgery, Samsung Medical Center, Sungkyunkwan University School of Medicine, Seoul, South Korea; Department of Thoracic Surgery, Chungbuk National University Hospital, Cheongju, Chungbuk South Korea

**Keywords:** Nontuberculous mycobacteria, Mycobacterium avium complex, Mycobacterium abscessus, Surgery

## Abstract

**Background:**

Outcomes of antibiotic treatment for lung disease caused by nontuberculous mycobacteria (NTM) are unsatisfactory. The role of adjunctive surgery in the treatment of NTM lung disease is still unclear.

**Methods:**

We conducted a retrospective review of 70 patients who underwent pulmonary resection for NTM lung disease from March 2007 to February 2013. All patients received recommended antibiotic treatment before and after the surgery.

**Results:**

A total of 70 patients underwent 74 operations. The median age of the patients was 50 years. Of the 70 patients, 45 (64%) had *Mycobacterium avium* complex infection (24 *M. intracellulare* and 21 *M. avium*) and 23 (33%) had *M. abscessus* complex infection (15 *M. abscessus* and 8 *M. massiliense*). Thirty-eight (54%) patients had the nodular bronchiectatic form and 28 (40%) had the fibrocavitary form of NTM lung disease. The indications for surgery were a poor response to drug therapy (n=52), remnant cavitary lesions and severe bronchiectasis (n=14), and hemoptysis (n=4). Preoperative sputum acid-fast bacilli staining results were positive in 44 (63%) patients, and sputum culture was positive in 54 (76%). The surgery included lobectomy or lobectomy plus segmentectomy (n=50, 68%), segmentectomy (n=11, 15%), pneumonectomy or completion pneumonectomy (n=8, 11%), bilobectomy or bilobectomy plus segmentectomy (n=4, 5%), and wedge resection (n=1, 1%). Postoperative complications occurred in 15 (21%) patients, including one postoperative death and bronchopleural fistula in 5 patients with the fibrocavitary form of the disease. A negative sputum culture was achieved and maintained in 57 (81%) patients.

**Conclusions:**

Although adjuvant pulmonary resection is associated with a relatively high complication rate, this procedure may provide a high level of treatment success for selected patients with NTM lung disease, such as those with a poor response to antibiotic treatment alone.

## Background

Nontuberculous mycobacteria (NTM) refer generally to mycobacteria other than Mycobacterium tuberculosis complex and *M. leprae*. The prevalence of lung diseases caused by NTM is increasing worldwide [1,2]. NTM Lung disease occurs commonly in structural lung disease, such as prior tuberculosis and bronchiectasis. Of the various species of NTM, *Mycobacterium avium* complex (MAC) such as *M. avium* and *M. intracellulare* and *M. abscessus* complex such as *M. abscessus* and *M. massiliense* are the most common causes of NTM lung disease [3,4].

A major therapeutic advance in the treatment of NTM lung disease was the introduction of the newer macrolides, clarithromycin and azithromycin, which have substantial *in vitro* and clinical activity against MAC and *M. abscessus* complex [1,2]. However, the treatment success rates for patients receiving combination antibiotic treatment for NTM lung disease are unsatisfactory. The reported success rates of medical treatment were approximately 40-55% in patients with MAC lung disease [5,6] and approximately 25-30% in those with *M. abscessus* lung disease [7-9]. Therefore, adjuvant resectional surgery could be considered in patients with intractable NTM lung disease predominantly localized to one lung and who can tolerate resectional surgery [10,11].

The increasing prevalence of NTM lung disease has been paralleled by increasing reports on the role of surgery since the introduction of the newer macrolides [12-25], suggesting more frequent use of thoracic surgery in the management of NTM lung disease. However, a majority of these reports were from limited centers in the Unites States and Japan, and the criteria for selecting the patients who may benefit from the operation remain controversial [10,26,27]. More data on the treatment outcomes of patients with NTM lung disease undergoing surgical resection in settings of various etiologic organisms will help determine the optimal role of adjuvant surgery.

The number of patients with NTM lung disease has continued to increase in South Korea [28-31]. We previously reported the treatment outcomes in 23 patients who underwent pulmonary resections for NTM lung disease [20]. This study reports our further experience of adjuvant surgical therapy in 70 patients with NTM lung disease.

## Methods

### Patients

We retrospectively reviewed the medical records of all patients who underwent adjunctive pulmonary resectional surgery for NTM lung disease at Samsung Medical Center (a 1,961-bed referral hospital in Seoul, Korea) between March 2007 and February 2013. During this period, a total of 70 patients underwent pulmonary resection for NTM lung disease. All patients met the diagnostic criteria for NTM lung disease [1].

NTM species were identified using polymerase chain reaction (PCR)-restriction fragment length polymorphism analysis or a PCR-reverse blot hybridization assay of the mycobacterial *rpoB* gene [32-34]. We classified chest radiography and high-resolution computed tomography (HRCT) scan findings as showing either fibrocavitary disease or nodular bronchiectatic disease. When the disease did not belong to either the fibrocavitary form or the nodular bronchiectatic form, it was deemed unclassifiable [35-38].

This study was approved by the Institutional Review Board of Samsung Medical Center to review and publish information obtained from patient records (IRB No. 2014-07-171). Informed consent was waived for the use of patient medical data and patient information was anonymized and de-identified prior to analysis.

### Preoperative antibiotic treatment

All patients with NTM lung disease received the standard combination antibiotic therapy according to the American Thoracic Society guidelines [1]. For treating MAC lung disease, patients received antibiotic therapy consisting of oral macrolide (clarithromycin or azithromycin), ethambutol, and rifampin. When necessary, streptomycin injection was added to the regimen in patients with severe MAC lung disease [35,39,40].

Patients with *M. abscessus* lung disease were treated with oral macrolide (clarithromycin or azithromycin) and/or oral fluoroquinolone (ciprofloxacin or moxifloxacin) in combination with parenteral antibiotics, including amikacin and cefoxitin (or imipenem), for the first 4 weeks of hospitalization [41]. For patients with *M. massiliense* lung disease, the same antibiotic regimens were used between March 2007 and September 2010. After October 2010, the initial duration of parenteral antibiotic therapy was reduced from 4 weeks to 2 weeks in patients with *M. massiliense* lung disease [42] because the treatment outcomes of antibiotic therapy were recognized to be superior in patients with *M. massiliense* lung disease than in those with *M. abscessus* lung disease [8,43,44]. The same oral regimens were used after discharge in patients with *M. abscessus* complex lung disease.

### Surgical treatment

Patients were selected for surgery based on consensus by medical and surgical specialists. In general, indications for pulmonary resection included (a) a poor response to medical therapy, (b) remnant cavitary lesions and/or severe focal bronchiectasis, and (c) development of complications such as massive hemoptysis [20]. Although there is no standard definition for NTM lung disease treatment failure, experts recommend that treatment failure be defined as failure to convert sputum to culture negative after at least 6 months of recommended therapy [10,22]. Therefore, we carefully selected patients who would benefit from resectional surgery, usually after at least 6 months of antibiotic treatment if these patients had not undergone previous treatment for NTM lung disease. However, we considered surgery more actively in some patients even before they completed 6 months of antibiotic therapy, if the patients had recurrent NTM lung disease with a history of previous treatment or had been transferred to our hospital because of persistent positive culture despite several months of antibiotic therapy. Surgical candidates had to have sufficient pulmonary function (≥40% of predictive postoperative forced expiratory volume in one second (FEV_1_) [45] to tolerate resection and a localized lesion with a large bacterial burden. For patients with bilateral lesions, the area with the larger bacterial burden was resected, and the remaining lesion with the smaller bacterial burden in the ipsilateral or contralateral lung was controlled with medical therapy. If treatment was ineffective, additional surgery was considered.

The standard preoperative work-up included chest radiography, chest HRCT, pulmonary function tests, quantitative lung perfusion scan, electrocardiogram, echocardiography, and bronchoscopy. Surgery was performed under general anesthesia using a double-lumen endobronchial tube. For patients in whom dense pleural adhesions were anticipated, such as those with apical fibrocavitary disease, we performed a posterolateral thoracotomy. For patients in whom dense pleural adhesions were not anticipated, such as those with middle lobe and lingular bronchiectatic disease, video-assisted thoracoscopic surgery was performed [26].

The same oral antibiotic regimens (oral macrolide, ethambutol, and rifampin for MAC lung disease and oral macrolide and/or oral fluoroquinolone for *M. abscessus* complex lung disease) were maintained after surgical resection and continued for at least 12 months after sputum culture conversion, defined as three consecutive negative sputum cultures.

### Statistical analyses

The data are presented as medians and interquartile ranges (IQR) for continuous variables and as numbers (percentages) for categorical variables. The data were compared using the Mann–Whitney *U* test for continuous variables and Pearson’s *χ*^2^ test or Fisher’s exact test for categorical variables. All statistical analyses were performed using PASW Statistics 20 (SPSS Inc., Chicago, IL).

## Results

### Baseline characteristics

The patient group included 42 females (60%) and 28 males (40%), with a median age of 50 years (IQR, 43–58 years). The median body mass index was 20.8 kg/m^2^ (IQR, 19.7-22.5 kg/m^2^). Fifty-one (73%) patients were nonsmokers. Six patients had recurrent NTM lung disease with a history of previous treatment of NTM lung disease and 15 patients were transferred to our hospital because of persistent positive culture despite several months of antibiotic therapy. Four patients had a history of previous pulmonary resection for pulmonary tuberculosis (n=2) or NTM lung disease (n=2).

None of the patients tested positive for human immunodeficiency virus. Median preoperative forced vital capacity was 2.82 L (IQR, 2.40-3.37 L), and forced expiratory volume in one second was 2.19 L (IQR 1.73-2.62 L) (Table 1).

**Table 1:** **Baseline characteristics of 70 patients**

**Characteristic**	**No. (%) or median (IQR)**
Age, years	50 (43–58)
Sex, male	28 (40)
Body mass index, kg/m^2^	20.8 (19.7-22.5)
Non-smoker	51 (73)
Underlying disease	
History of pulmonary TB	45 (64)
Previous therapy for NTM lung disease	6 (9)
Receiving antibiotic therapy at the time of transfer	15 (21)
Previous lung resection	4 (6)
Diabetes mellitus	5 (7)
Etiology	
*Mycobacterium avium* complex	45 (64)
*M. intracellulare* / *M. avium*	24 / 21
*Mycobacterium abscessus* complex	23 (33)
*M. abscessus* / *M. massiliense*	15 / 8
Mixed infection	2 (3)
Type of disease	
Nodular bronchiectatic	38 (54)
Fibrocavitary	28 (40)
Unclassifiable	4 (6)
Preoperative sputum examinations	
Positive AFB stain	44 (63)
Positive AFB culture	54 (76)
Preoperative pulmonary function test	
FVC (L)	2.82 (2.40-3.37)
FVC, % predicted	80 (70–95)
FEV_1_ (L)	2.19 (1.73-2.62)
FEV_1_, % predicted	81 (63–92)

The data are presented as medians and interquartile ranges or as numbers (%).

IQR, interquartile range; TB, tuberculosis; AFB, acid-fast bacilli; FEV_1,_ forced expiratory volume in one second; FVC, forced vital capacity.

Of the 70 patients, 45 (64%) had MAC infection (*M. intracellulare* in 24 and *M. avium* in 21), 23 (33%) had *M. abscessus* complex infection (*M. abscessus* in 15 and *M. massiliense* in 8), and two (3%) had mixed MAC and *M. abscessus* infection. The preoperative sputum smear and culture results were positive in 44 (63%) and 54 (76%) patients, respectively. Based on the chest radiography and HRCT findings, 38 (54%) patients had the nodular bronchiectatic form and 28 (40%) had the fibrocavitary form of NTM lung disease (Table 1).

### Medical and surgical treatment

The indications for surgery included a poor response to medical therapy (n=52, 74%), remnant cavitary lesions and/or severe focal bronchiectasis (n=14, 20%), and massive hemoptysis during antibiotic therapy (n=4, 6%). Standardized combination antibiotic therapy was administered preoperatively in all patients. The median duration of preoperative antibiotic therapy was 8.3 months (IQR, 3.8-14.7 months). In 21 patients who had recurrent NTM lung disease or who were transferred to our hospital because of persistent positive culture despite long-term antibiotic treatment, the duration of antibiotic treatment in our hospital was a median of 6.7 months (IQR 2.6-8.2 months), which was shorter compared with the 49 patients who did not have a history of previous NTM lung disease and started antibiotic therapy in our hospital (median 11.0 months, IQR 4.9-18.2 months, P=0.018).

Among 45 patients with MAC lung disease, streptomycin injection was added in 21 patients with the fibrocavitary form and nine patients with nodular bronchiectatic or unclassifiable form who had cavitary lesions. However, three patients discontinued streptomycin due to side effects such as skin rash or perioral tingling sensation. Among 15 patients with the nodular bronchiectatic or an unclassifiable form who did not have cavitary lesions, six patients received streptomycin injection at the discretion of the attending physician.

The 70 patients underwent 74 pulmonary resections (Table 2). Open thoracotomy, minithoracotomy with video assistance, and video-assisted thoracoscopic surgery without minithoracotomy were performed in 47 (64%), 1 (1%), and 26 (35%) resections, respectively. Bronchial stump reinforcement was performed in two patients (one patient receiving lobectomy plus segmentectomy and one receiving bilobectomy).

**Table 2:** **Type of lung resection and postoperative complications**

**Type of resection**	**No. (%) of lung resection (n=74)**	**Complications (n=15)**	
	**[Right/Left]**	**After right-sided resection**	**After left-sided resection**
Pneumonectomy	5 (7%) [2/3]	Postoperative mortality (1)	None (0)
Completion pneumonectomy	3 (4%) [1/2]	Empyema (1)	Vocal cord palsy (1)
Bilobectomy plus segmentectomy	1 (1%) [1/0]	BPF, empyema, wound dehiscence (1)*	None (0)
Bilobectomy	3 (4%) [3/0]	none (0)	None (0)
Lobectomy plus segmentectomy	8 (11%) [4/4]	BPF (1), wound dehiscence (1)	BPF (1), empyema (1)
Lobectomy	42 (57%) [25/17]	BPF (1), wound dehiscence (1), Pneumonia (1)	Wound dehiscence (1), Pericarditis (1), atrial fibrillation (1)
Segmentectomy	11 (15%) [4/7]	None (0)	BPF (1)
Wedge resection	1 (1%) [1/0]	None (0)	None (0)

BPF, bronchopleural fistula.

*Postoperative bronchopleural fistula, empyema, and wound dehiscence developed in the same patient.

### Postoperative complications

There were no intraoperative deaths. There was one postoperative death during hospitalization; a 57-year-old man with *M. intracellulare* lung disease died of pneumonia and acute respiratory failure 79 days after undergoing right pneumonectomy. Postoperative complications occurred in 15 patients (21%), including one postoperative death (Table 2). Among four patients who underwent the second surgery, one patient developed bronchopleural fistula after the first surgery. Postoperative complication rates did not differ between patients with preoperative positive sputum culture results (19% [13/54]) and those with preoperative negative sputum culture results (13% [2/16], P=0.322). Postoperative complication rates were slightly low after video-assisted thoracoscopic surgery (15% [4/27]) compared with those after open thoracotomy (23% [11/47]); however, this was not a significant difference (P=0.376).

All five patients who developed bronchopleural fistula had the fibrocavitary form of NTM lung disease (two in MAC and three in *M. abscessus* complex). Preoperative sputum smear and culture were positive in four (80%) and five (100%) patients, respectively. The type of resection included bilobectomy plus segmentectomy (n=1), lobectomy plus segmentectomy (n=2), lobectomy (n=1), and segmentectomy (n=1).

Among 45 patients with MAC lung disease, the postoperative complication rate was high (21% [7/33]) in 33 patients who received streptomycin injection compared with 12 patients who did not receive streptomycin injection (8% [1/12]); however, this difference was not statistically significant (P=0.318). Among 23 patients with *M. abscessus* complex lung disease, 19 patients received perioperative parenteral antibiotics such as amikacin and cefoxitin (or imipenem) for a median of 28 days (IQR 25–33 days). Postoperative complication rates did not differ between patients who received perioperative parenteral antibiotics (26% [5/19]) and those who did not (25% [1/4], P=0.957).

### Treatment outcomes

Postoperative antibiotic therapy was administered to all patients, with a median treatment time of 12.7 months (IQR, 12.0 to 18.2 months). The same oral antibiotic regimens (oral macrolide, ethambutol, and rifampin for MAC lung disease and oral macrolide and/or oral fluoroquinolone for *M. abscessus* complex lung disease) were used after surgical resection. Of 70 patients, 57 (81%) achieved sputum culture conversion. No relapse occurred in these patients during a median follow-up period of 27.3 months (IQR, 19.8 to 36.9 months).

Among 45 patients with MAC lung disease, the sputum culture conversion rate was low (82% [27/33]) in the 33 patients who received streptomycin injection compared with the 12 patients who did not receive streptomycin injection (100% [12/12]); however, this difference was not statistically significant (P=0.472). In 23 patients with *M. abscessus* complex lung disease, sputum culture conversion rates were not different between patients who received perioperative parenteral antibiotics (63% [12/19]) and those who did not (100% [4/4], P=0.957).

Among the four patients who underwent a second resectional surgery, three obtained successful sputum culture conversion after the second resection, while one patient failed to achieve sputum culture conversion. Late death occurred in one patient with *M. abscessus* lung disease at 53 months after surgery due to pneumonia and empyema associated with bronchopleural fistula.

### Comparisons between patients with MAC lung disease and those with M. abscessus complex lung disease

We compared preoperative baseline characteristics, type of surgery, and treatment outcomes between 45 patients with MAC lung disease and 23 with *M. abscessus* complex lung disease after exclusion of two patients with mixed infection. As shown in Table 3, the preoperative baseline characteristics did not differ between the two groups. Type of lung resection and the proportion of patients who underwent surgery using a thoracoscopic approach were not different between the two groups. Although the total postoperative complication rates did not differ between the two groups, development of wound dehiscence was more frequent in patients with *M. abscessus* complex compared with MAC patients (13% [3/23] vs. 0% [0/45], P=0.035). Although sputum culture conversion rates were higher in MAC patients (87% [39/45]) compared with *M. abscessus* complex patients (70% [16/23]), this difference was not statistically significant (P=0.111, Table 3).

**Table 3:** **Baseline characteristics of 45 patients with MAC lung disease and 23 with** ***M. abscessus*** **complex lung disease**

**Characteristic**	**MAC (n=45)**	***M. abscessus*** **complex (n=23)**	**P value**
Age, years	50 (43–61)	50 (42–55)	0.241
Sex, male	21 (50)	6 (26)	0.101
Type of disease			0.181
Nodular bronchiectatic	21 (47)	16 (70)	
Fibrocavitary	21 (47)	7 (30)	
Unclassifiable	3 (7)	0	
Preoperative sputum examinations			
Positive AFB stain	26 (58)	17 (74)	0.192
Positive AFB culture	35 (78)	17 (74)	0.722
Indications of surgery			0.812
Poor response to medical therapy	34 (76)	16 (70)	
Remnant destroyed lesions	8 (18)	6 (26)	
Massive hemoptysis	3 (7)	1 (4)	
Type of resection*			0.292
Pneumonectomy	4 (9)	1 (4)	
Completion pneumonectomy	3 (6)	0	
Bilobectomy plus segmentectomy	0	1 (4)	
Bilobectomy	1 (2)	2 (8)	
Lobectomy plus segmentectomy	3 (6)	4 (16)	
Lobectomy	26 (55)	15 (60)	
Segmentectomy	9 (19)	2 (8)	
Wedge resection	1 (2)	0	
Surgical approach			0.596
Open thoracotomy	29 (62)	17 (68)	
Thoracoscopic approach^†^	18 (38)	9 (32)	
Postoperative complications			0.529
Postoperative mortality	1 (2)	0	1.000
Bronchopleural fistula	2 (4)	3 (13)	0.334
Wound dehiscence	0	3 (13)	0.035
Empyema	1 (2)	2 (9)	0.257
Pneumonia	1 (2)	0	1.000
Vocal cord palsy	1 (2)	0	1.000
Pericarditis	1 (2)	0	1.000
Atrial fibrillation	1 (2)	0	1.000
Sputum culture conversion	39 (87)	16 (70)	0.111

The data are presented as medians and interquartile ranges or as numbers (%).

MAC, Mycobacterium avium complex; TB, tuberculosis; AFB, acid-fast bacilli.

*Forty-five MAC patients underwent 47 operations, and 23 M. abscessus complex patients underwent 25 operations.

^†^One patient underwent surgery using the thoracoscopic approach with minithoracotomy.

### Comparisons between patients with nodular bronchiectatic form and those with fibrocavitary form of NTM lung disease

We compared preoperative baseline characteristics, type of surgery, and treatment outcomes for 38 patients with the nodular bronchiectatic form and 28 with the fibrocavitary form of NTM lung disease after exclusion of four patients with unclassifiable forms. Patients with the nodular bronchiectatic form were more likely to be female (76% [29/38] vs. 39% [11/28], P=0.002) and non-smokers (84% [32/38] vs. 61% [17/28], P=0.031), and also had lower pre-operative sputum positive culture rates (66% [25/38] vs. 89% [25/28], P=0.028) compared with patients with the fibrocavitary form of NTM lung disease (Table 4).

**Table 4:** **Baseline characteristics of 38 patients with nodular bronchiectatic and 28 with fibrocavitary nontuberculous mycobacterial lung disease**

**Characteristic**	**Nodular bronchiectatic (n=38)**	**Fibrocavitary (n=28)**	**P value**
Age, years	52 (43–60)	51 (44–60)	0.943
Sex, male	9 (24)	17 (61)	0.002
Etiology			0.195
*M. avium* complex	21 (55)	21 (75)	
*M. abscessus* complex	16 (42)	7 (25)	
Mixed infection	1 (3)	0	
Preoperative sputum examinations			
Positive AFB stain	20 (53)	20 (71)	0.122
Positive AFB culture	25 (66)	25 (89)	0.028
Indications of surgery			0.254
Poor response to medical therapy	26 (68)	22 (79)	
Remnant destroyed lesions	8 (21)	6 (21)	
Massive hemoptysis	4 (11)	0 (0)	
Type of resection*			0.465
Pneumonectomy	2 (5)	3 (10)	
Completion pneumonectomy	1 (2)	2 (7)	
Bilobectomy plus segmentectomy	0	1 (3)	
Bilobectomy	3 (7)	0	
Lobectomy plus segmentectomy	4 (10)	3 (10)	
Lobectomy	25 (61)	14 (48)	
Segmentectomy	5 (12)	6 (21)	
Wedge resection	1 (2)	0	
Surgical approach			0.012
Open thoracotomy	22 (54)	24 (83)	
Thoracoscopic approach^†^	19 (46)	5 (17)	
Postoperative complications			0.029
Postoperative mortality	0	1 (4)	0.424
Bronchopleural fistula	0	5 (18)	0.011
Wound dehiscence	1 (3)	2 (7)	0.570
Empyema	2 (5)	1 (4)	1.000
Pneumonia	0	1 (4)	0.424
Vocal cord palsy	0	1 (4)	0.424
Pericarditis	0	0	1.000
Atrial fibrillation	1 (3)	0	1.000
Sputum culture conversion	30 (79)	23 (82)	0.747

The data are presented as medians and interquartile ranges or as numbers (%).

TB, tuberculosis; AFB, acid-fast bacilli.

*Thirty-eight patients with the nodular bronchiectatic form underwent 41 operations, and 28 patients with the fibrocavitary form underwent 29 operations.

^†^One patient underwent surgery using a thoracoscopic approach with minithoracotomy.

The proportion of thoracoscopic surgery was significantly higher in patients with the nodular bronchiectatic form compared to those with the fibrocavitary form of NTM lung disease (46% [19/41] vs. 17% [5/29], P=0.012), although type of lung resection did not differ between the two groups (Table 4). Among postoperative complications, bronchopleural fistula developed more frequently in patients with fibrocavitary lung disease compared to those with the nodular bronchiectatic form of NTM lung disease (18% [5/28] vs. 0%, P=0.011). However, sputum culture conversion rates were not different between the two groups (Table 4).

## Discussion

This study evaluated the treatment outcomes in 70 patients who underwent adjunctive pulmonary resections for NTM lung disease. The most common indication for surgery was a poor response to antibiotic therapy. Successful treatment outcomes were achieved in more than 80% of patients in our study, although the postoperative complication rate was relatively high. The present study is the second-largest case series to evaluate the role of adjunctive resectional surgery for NTM lung disease after reports by a group from Denver in the United States [21,23,24]. Although postoperative outcomes and complications vary by type of surgery and severity of disease, our study results are in agreement with many of the studies published in the past 20 years (Table 5).

**Table 5:** **Treatment outcomes of resectional surgery for NTM lung disease in previous reports published between 1994 and 2014**

**Author, year, reference**	**Study period**	**No. of Patients (No. of Resections)**	**Common etiology (No.)**	**Sputum culture conversion rate (%)**	**Long-term relapse rate (%)**	**Postoperative mortality/morbidity (%)**
Pomerantz, 1996 [12]	1983-1996	13 (15)	MAC (12)	100	8	0/8
Ono, 1997 [13]	1991-1996	8 (8)	MAC (8)	100	13	0/0
Shiraishi, 1998 [14]	1979-1996	33 (33)	MAC (33)	94	6	0/15
Nelson, 1998 [15]	1989-1997	28 (28)	MAC (28)	79	4	7/29
Shiraishi, 2002 [16]	1993-2001	21 (21)	MAC (21)	100	10	0/29
Shiraishi, 2004 [17]*	1983-2002	11 (11)	MAC (10), MABC (1)	100	9	18/36
Sherwood, 2005 [18] ^†^	1994-2003	26 (26)	MAC (15), MABC (1)	82	0	23/46
Watanabe, 2006 [19]	1990-2005	22 (25)	MAC (22)	100	5	0/5
Koh, 2008 [20]	2002-2006	23 (23)	MAC (10), MABC (12)	91	0	4/35
Mitchell, 2008 [21]	1983-2006	236 (265)	MAC (189), MABC (32)	NA	NA	3/12
van Ingen, 2010 [22]	2000-2009	8 (8)	MAC (7)	88	0	13/50
Yu, 2011 [23]	2004-2009	134 (172)	MAC (118), MABC (14)	84	7	0/7
Jarand, 2011 [7]	2001-2008	24 (29)	MABC (24)	57	NA	17/25
Mitchell, 2012 [24]	2004-2010	171 (212)	MAC (147), MABC (36)	NA	NA	0/9
Shiraishi, 2013 [25]	2007-2011	60 (65)	MAC (55), MABC (3)	100	3	0/12
This study	2007-2013	70 (74)	MAC (45), MABC (23)	81	0	1/20

NTM, nontuberculous mycobacteria; MAC, Mycobacterium avium complex; MABC, Mycobacterium abscessus-massiliense complex; NA, not available.

*All patients underwent pneumonectomy.

^†^All patients underwent completion pneumonectomy.

NTM lung disease has become a significant health problem. However, NTM lung disease remains difficult to treat with medication alone, although the introduction of newer macrolides has improved the outcome of its medical treatment [1,2]. Therefore, pulmonary resection surgery has been advocated for selected patients who have localized disease and are able to tolerate resection to remove gross lesions that contain large numbers of bacilli [10,11].

Regarding the treatment of NTM lung disease, the American Thoracic Society guidelines recommend that the initial therapy for patients with MAC lung disease consist of a minimum three-drug regimen of oral macrolide, ethambutol, and rifampin [1]. In addition, intermittent streptomycin or amikacin injection for the first 2 to 3 months of therapy is recommended to treat extensive disease [1]. Nevertheless, the success rates of these medical treatments are not satisfactory [5,6], and the relapse rate in patients with sputum conversion at the completion of medical treatment is high [46].

For the treatment of *M. abscessus* complex lung disease, combined intravenous antibiotic therapy including amikacin and cefoxitin (or imipenem) in addition to the oral antibiotics was recommended [1]. However, the antibiotic treatment of lung disease caused by *M. abscessus* is usually unsuccessful [7-9]. Therefore, surgery is typically recommended for patients with localized lung disease who can withstand lung resection after an initial period on antimicrobials to reduce the microbial burden [1].

Therefore, the patients whose disease was mainly localized to one lung and who could tolerate pulmonary resection were considered for surgery [10,11]. In this study, we were able to assess the surgical outcomes of 70 patients with NTM lung disease. A majority of our patients (76%) had preoperative culture-positive sputum for NTM despite treatment with long-term antibiotic therapy. However, postoperative negative sputum culture conversion was achieved and maintained in 81% of patients and no relapse occurred in these patients during the follow-up period. These results indicate that pulmonary resection can play an important role in achieving a better outcome in selected patients, especially those in whom medical therapy had failed or who have a remnant lesion with a high possibility of relapse.

The optimal duration of antibiotic therapy before surgery remains controversial [14,26,27]. In the present study, the median duration of preoperative antibiotics was 8.3 months. Patients are expected to show clinical improvement within 3–6 months and for the sputum to convert to negative within 12 months on antibiotic treatment regimens [1]. Patients are considered to be treatment failures if they have not had a response (microbiologic, clinical or radiographic) after 6 months of appropriate therapy or have not achieved sputum conversion to culture-negative after 12 months of appropriate therapy [1,10]. Therefore, failure to respond in these time periods should prompt investigation of the potential role of surgery.

The optimal duration of antibiotic therapy after surgery also remains unclear [14,26,27]. The median duration of postoperative antibiotic therapy was 12.7 months in patients with successful negative sputum culture conversion in our study. The guidelines stated that patients with NTM lung disease should be treated until achieving a negative sputum culture on therapy for 12 months [1]. This requirement means that postoperative antibiotic therapy for 12 months would be appropriate if sputum conversion is achieved after surgery.

Despite the favorable treatment outcomes, postoperative complications were relatively common (21%) with NTM lung disease in our study. This rate was similar to those of previous studies that reported a high incidence of postoperative morbidity (Table 5). Among various postoperative complications, this study found that wound dehiscence occurred more frequently in patients with *M. abscessus* complex lung disease compared to those with MAC lung disease. This difference may be related to the fact that *M. abscessus* complex is naturally resistant to more antibiotics in clinical use [9,47].

Bronchopleural fistula developed in five patients in our study population; all of these patients had the fibrocavitary form of NTM lung disease with severe pleural adhesions and did not receive bronchial stump reinforcement. We did not apply bronchial stump reinforcement routinely in the study period, particularly in the early phase or in cases with healthy-looking bronchus. The goal of case-by-case application of bronchial stump reinforcement was to avoid extra surgical procedures and reduce operation time. For minimally invasive procedures such as video-assisted thoracoscopic surgery, the avoidance of unnecessary procedures is thought to be important. We had quite good surgical outcomes in a previous study of multidrug resistant and extensively drug-resistant tuberculosis patients [48]. In that study, there were no cases of bronchopleural fistula, although only 12 of 72 patients (17%) received bronchial stump reinforcement [48]. Considering the relatively frequent development of bronchopleural fistula in NTM patients, however, the present study suggested that application of bronchial stump reinforcement should be considered carefully in patients who received resectional surgery for the fibrocavitary form of NTM lung disease. Regarding potentially severe postoperative complications, especially in patients with *M. abscessus* complex lung disease or the fibrocavitary form of NTM lung disease, surgery should be performed at centers employing thoracic surgeons who have considerable experience with this type of surgery [1].

Most of the adjunctive surgical procedures for NTM lung disease included lobectomy or pneumonectomy other than minimal pulmonary resection. In the current study, we performed 11 segmentectomies (15%) and one wedge resection (1%) for patients who had limited lesions in each segment of a lobe or marginal pulmonary function because of underlying pulmonary disease. In addition, 36% of resections were performed using video-assisted thoracoscopic surgery. Video-assisted thoracoscopic surgery has been more widely used in patients with NTM lung disease, especially the nodular bronchiectatic form, in recent years [23,24]. Minimal pulmonary resection and minimally invasive approaches should be considered for the preservation of lung parenchyma and lung function in selected patients who have a small and localized lesion below the segmental level of the lung.

As with all research, the present study is not without limitations. First, this retrospective study was conducted at a single referral center. Furthermore, the surgical patients were highly selected, and this selection could bias the beneficial outcome. Second, the duration of postoperative follow-up was relatively short, and this study did not assess the long-term outcomes, such as relapse rate after treatment completion. Long-term follow-up is needed to assess the true efficacy of adjunctive resection because relapse and reinfection may occur years after the completion of therapy.

## Conclusion

In conclusion, despite its relatively high surgical complication rate, NTM lung disease in patients with localized disease and who can tolerate resectional surgery might be considered for surgery if they have had a poor response to drug therapy or if they develop significant disease-related complications such as hemoptysis. Clinicians should carefully select those patients with NTM lung disease who can benefit from adjunctive resectional surgery.

## Abbreviations

HRCT: High-resolution computed tomography
IQR: Interquartile ranges
MAC: Mycobacterium avium complex
NTM: Nontuberculous mycobacteria
PCR: Polymerase chain reaction
